# Examination of Impact of NBTIs on Commercial Power P-Channel VDMOS Transistors in Practical Applications

**DOI:** 10.3390/mi17010052

**Published:** 2025-12-30

**Authors:** Danijel Danković, Emilija Živanović, Nevena Veselinović, Dunja Đorđević, Marija Petrović, Lana Tasić, Miloš Marjanović, Sandra Veljković, Nikola Mitrović, Vojkan Davidović, Goran Ristić

**Affiliations:** Faculty of Electronic Engineering, University of Niš, Aleksandra Medvedeva 4, 18000 Niš, Serbia; emilija.zivanovic@elfak.ni.ac.rs (E.Ž.); nevena.veselinovic@elfak.rs (N.V.); dunja.djordjevic@elfak.rs (D.Đ.); marija.petrovic@elfak.rs (M.P.); lana.tasic@elfak.rs (L.T.); milos.marjanovic@elfak.ni.ac.rs (M.M.); sandra.veljkovic@elfak.ni.ac.rs (S.V.); nikola.i.mitrovic@elfak.ni.ac.rs (N.M.); vojkan.davidovic@elfak.ni.ac.rs (V.D.); goran.ristic@elfak.ni.ac.rs (G.R.)

**Keywords:** VDMOS, NBTI, self-heating effects, CMOS inverter, threshold voltage shift

## Abstract

In this paper, the impact of negative bias temperature instabilities (NBTIs) on commercial power p-channel Vertical Double-Diffused MOS (VDMOS) transistors from the standpoint of practical applications was analyzed. The effects of NBTI are one of the main reliability concerns for this type of device, so it is necessary to investigate how these effects influence various applications. A series of experiments were carried out including negative bias temperature stressing, infra-red thermographic recording and circuit characterization, with the goal of evaluating the effects of negative bias temperature stressing on the self-heating of samples in load-driving circuits operating with higher currents and circuit performance of a CMOS inverter circuit containing the examined samples. The findings suggest that negative bias temperature stressing-induced threshold voltage shift directly affects increased self-heating in load-driving circuits and that it also directly affects transfer and dynamics characteristics in CMOS inverters.

## 1. Introduction

Through research and development, power VDMOS (Vertical Double-Diffused Metal Oxide Semiconductor) transistors have been proposed as devices suitable for multiple practical applications in different industries. Their wide range of utilization includes applications in the automotive industry and aerospace industry, as well as in switching power supplies and motor drivers [1,2,3]. This vast scope of use cases enables application in different types of circuits and various environments (presence of different types of irradiation or extreme temperatures). Although there are many reports in the literature investigating the reliability of VDMOS devices, constant expansion of application areas in different industry branches demands continuous attention to reliability considerations, such as degradation mechanisms, operating limitations and lifetime estimations [2,4,5,6,7].

The main reliability concerns regarding power VDMOS devices are BTIs (bias temperature instabilities), SHEs (self-heating effects), HCEs (Hot Carrier Effects) and TDDB (Time-Dependent Dielectric Breakdown) [4,8,9]. These effects represent the main parametric failure mechanisms in practical applications.

### 1.1. Effect of Negative Bias Temperature Instabilities

One of the most serious effects that alters the parameters of VDMOS transistors is represented by bias temperature instabilities (BTIs). Depending on whether the bias is negative or positive, two different groups of effects (NBTIs and PBTIs) are reported in the literature; the effects of NBTI on p-channel devices are the most significant ones [4,5]. In many studies devoted to understanding NBTIs, it has been found that this phenomenon is a result of the generation of oxide-trapped charge (*N*_ot_) and interface traps (*N*_it_), which occur during the operation of devices. NBTIs have been found to occur mostly in p-channel devices operated under negative gate oxide fields in the range 2–6 MV/cm and at elevated temperatures (100–250 °C) [5,10,11].

**Figure 1:**
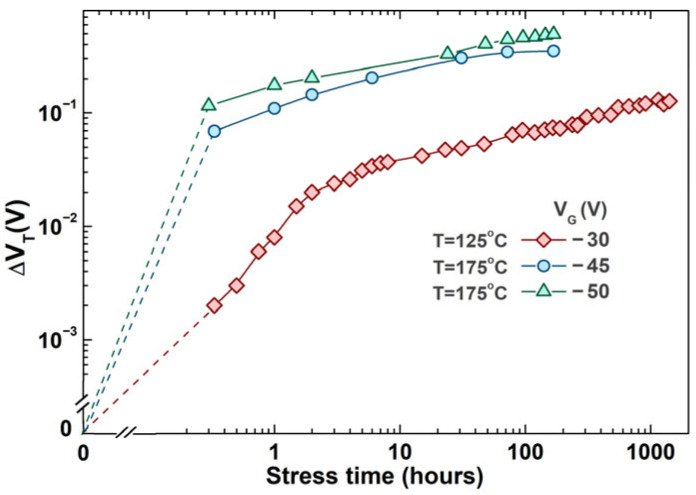
Threshold voltage shift in p-channel power VDMOSFET subjected to NBT stress under different stressing conditions.

In p-channel devices, NBTI leads to an increase in the absolute value of the threshold voltage, which is one of the crucial parameters of the MOS transistor, and an increase in drain-source resistance when the device is in an on-state (*R*_DS(ON)_); it also results in a decrease in the device transconductance (*g*_m_) and the absolute drain current (*I*_Dsat_). Even though the first publication describing the effects of NBTI from Jeppson and Svenson dates back to 1977, scaling and miniaturization of devices led to an increasing number of studies on this topic in the previous decade [10,11,12]. The main degradation mechanisms still remain a subject of debate [11], while many different analytical and compact models covering various transistor structures have been developed [6,13,14,15]. Many reports in the literature, including this one, are focused on investigating the physical mechanisms and responsible processes that occur during negative bias temperature stressing (NBTS) in VDMOS devices [4,5,7,16,17]. Effects are typically observed under accelerated stress conditions, meaning that they are under elevated gate voltage and temperature values. In previous decades, our research included a great number of different combinations of gate voltages and temperatures, each producing corresponding threshold voltage shifts. Experiments have been carried out for combinations of five different gate voltages (−30 V, −35 V, −40 V, −45 V and −50 V) and three different temperatures (125 °C, 150 °C and 175 °C). Part of these previous results, which show Δ*V*_T_ for the conditions producing the largest shift (*V*_G_ = −50 V, T = 175 °C) and the smallest shift (*V*_G_ = −30 V, T = 125 °C), as well as the most frequently used stress conditions (*V*_G_ = −45 V, T = 175 °C), are presented in Figure 1. It should be noted that an investigation of pulsed NBT stressing for different frequency and duty cycle values, under the described gate voltages and temperature conditions, has been reported in our previous publications [18,19], with experiment durations from 24 h to 2000 h [18,19,20,21].

Figure 1 shows the threshold voltage shift in devices subjected to NBT stress under selected conditions. It should be emphasized that the group stressed at 125 °C was exposed to NBT stress for 1416 h, whereas the groups stressed at 175 °C were exposed to NBT stress for 168 h. A pronounced difference in threshold voltage shift is observed between the stress conditions at 125 °C and 175 °C, with significantly larger shifts recorded at 175 °C. It is also clearly visible that the applied gate voltage has a substantial influence on the threshold voltage variation. Specifically, devices exposed to a gate voltage of −50 V exhibit larger threshold voltage shifts compared to those stressed at −45 V under the same temperature conditions.

Furthermore, in the initial phase of NBT stress, differences in threshold voltage shift rates can be observed between devices stressed at the same temperature but under different stress voltages. The data show that the rate of threshold voltage shift is higher for devices exposed to the higher gate voltage (−50 V) compared to those stressed at lower stress voltages (−45 V).

Recent reports also suggest that the effects of NBTI can have a direct impact on the practical applications of VDMOS devices [2,3,5,16]. Their superior switching characteristics, which allow operation in the megahertz range, tend to suffer from NBTI-induced pulse narrowing. In switching applications, the p-channel VDMOS operates as a switch, where turning on the transistor (with negative voltage in p-channel devices) leads to channel inducing and allows for conductance of current. Turning off the transistor thereby disallowing the conductance of current. The turning on/off of the transistor is controlled with the signal that is being fed to the gate of the transistor.

**Figure 2:**
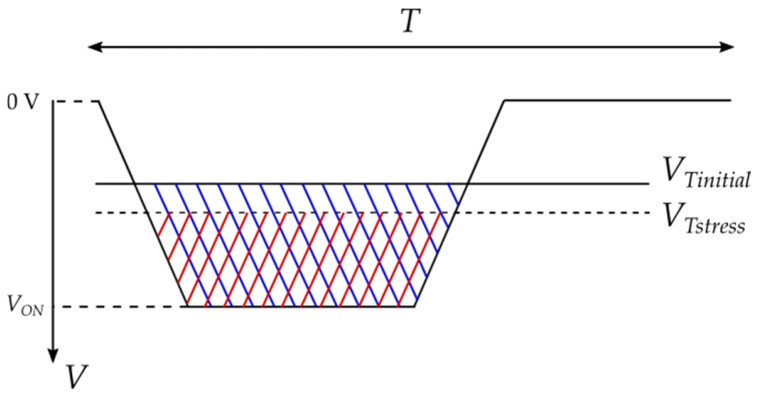
Illustration of NBTI-induced pulse narrowing and reduced operation area during on-time of one period.

Continuous operation of the devices leads to eventual degradation; a change in the threshold voltage is one of the most significant changes. During longer operation intervals, the controlling signal, which is being generated from some external circuit, usually keeps its initial characteristics, while the characteristics of the transistor alter. This leads to some limitations in operations and some unwanted effects, which are considered in the literature as NBTI pulse narrowing [3]. An illustration depicting this effect is shown in Figure 2.

When the threshold voltage is changed (increased in absolute value), the transistor will turn on later than expected, meaning later than it will turn on if the threshold voltage sustains its initial value. Therefore, the transistor will sustain the generated channel between the drain and the source terminals for a shorter interval of time than expected. This also shortens the interval of the conduction of current, which can make a direct impact on some of the applications. These effects are more emphasized with longer *t*_rise_ and *t*_fall_ intervals, whereas in practical application of the device, longer transition times are typical. Conclusions from the literature suggest that further examination is needed to assess the impact of NBTI in direct practical applications [2,3,5].

### 1.2. Self-Heating Effects

**Figure 3:**
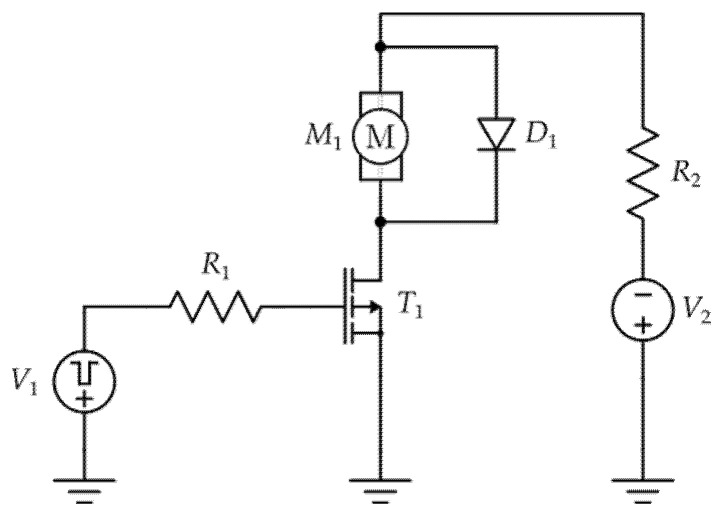
An example of the Basic DC motor driving circuit with p-channel power VDMOSFET operating as a high-end switch [23].

Besides NBTI, the significant impact of the parameters of VDMOS devices is caused by self-heating effects. Power p-channel devices are able to conduct relatively high currents (for example, commercial power p-channel VDMOSFET IRF9520 (Vishay, Malvern, PA, USA) can conduct a maximum drain current of 6.8 A, as stated in the datasheet [22]). This characteristic is widely used in motor driver applications. While there are many different motor driving circuits reported in the literature, one of the most basic circuits is presented in Figure 3 [23].

When conducting higher currents, the power dissipation of the device increases, leading to an increase in self-heating. There are several investigations reporting that excessive self-heating wears out power VDMOS devices and degrades their parameters [5,23,24,25,26]. A combination of self-heating and higher ambient temperature can lead to the parametric failure of the device.

One of the parameters that deviates due to either aging or accelerated NBT stress is the change in the turn-on resistance (*R*_DS(ON)_). One of the consequences of *R*_DS(ON)_ increase is increased channel resistance (*R*_ch_), which affects power dissipation during the change between the turned-on and turned-off state. Previous investigations suggested that, even though the area of operation is reduced after threshold voltage shift, as is illustrated in Figure 2, self-heating of the devices is not necessarily reduced accordingly. This is caused by increased power dissipation during state changes.

**Figure 4:**
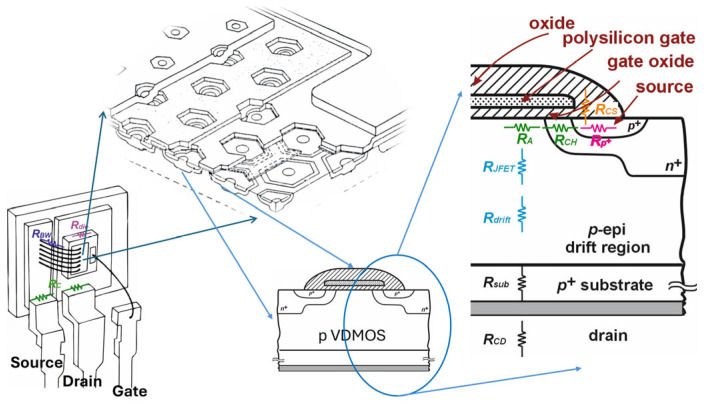
Illustration of cross-section of the commercial p-channel power VDMOSFET with marked internal resistances.

Figure 4 schematically illustrates the internal architecture of a power VDMOS transistor, consisting of hundreds of hexagonal unit cells, with two representative half-cells highlighted to emphasize the current flow distribution and the locations of the dominant resistances. The figure provides an expanded view of the structural regions that contribute to the overall on-state resistance of the device.

In addition to the resistances originating from the active regions, such as the channel, p+ source region, JFET and drift region, the total on-state resistance is also significantly influenced by elements located outside the chip but still within the TO-220 package. These include the bond wire resistance (*R*_BW_), bonding solder resistance (*R*_Die_) and contact resistance *R*_C_ [27]. Combined, these form the drain contact resistance (*R*_CD_). These packaging-related resistances become particularly relevant at high currents and elevated operating temperatures.

Therefore, many previous studies have concluded that more investigation is needed to better assess the self-heating effects in p-channel VDMOS devices and determine the effects of prior treatment on self-heating [5,24,25,26].

### 1.3. Impact on the Circuit Performance

Besides self-heating effects, additional attention should be given to the impact of NBT stressing on the performance of the circuits containing p-channel VDMOS devices. Although many studies have been performed with the goal of evaluating responsible physical mechanisms occurring during the operation and stressing of VDMOS devices, focus on only device-level degradation does not fully describe the impact of NBTI. When in circuit, the p-channel VDMOS device interacts with other devices, with power supplies and connections. This type of circuit-level reliability investigation is needed in order to faithfully reflect the impact of NBTI on circuit performance [28,29].

There are several reports in the literature targeting the impact of NBTI on circuit performance [28,29,30]. In most of the available studies, the authors investigate effects on rather simple circuits, such as a CMOS inverter [28,29,30,31,32]. As shown in Figure 2, NBT stress-induced degradation of a p-channel VDMOSFET comprising a CMOS inverter and NBTI-induced pulse narrowing directly affects the transient times of a CMOS inverter, as it would impact the transient times of even more complex circuits. Thorough analysis is needed in order to assess the impact of NBTI on circuit performance [28].

In order to tackle ongoing open questions regarding examination and impact of the NBTI on self-heating effects and circuit performance, a series of extensive experiment sessions have been carried out. The rest of the paper will present a detailed explanation, analysis of the experimental setup and the obtained results.

## 2. Experiment

The experimental investigation started with a large number of samples, analyzing different conditions to obtain results. Samples are commercial p-channel VDMOS devices IRF9520 with polysilicon gate technology and hexagonal cell geometry [22]. These devices, encapsulated in TO-220 packages, have a gate oxide thickness of approximately 100 nm. Maximum continuous drain current is 6.8 A, while the value of the threshold voltage is in the range from −2 V to −4 V. Although the threshold voltage lies within this range, previously conducted experiments have shown that identical stress conditions consistently produce the same threshold-voltage shifts, regardless of the initial value. The samples from this experiment had an initial value of *V*_T_ = 3 V ± 0.005 V.

**Figure 5:**
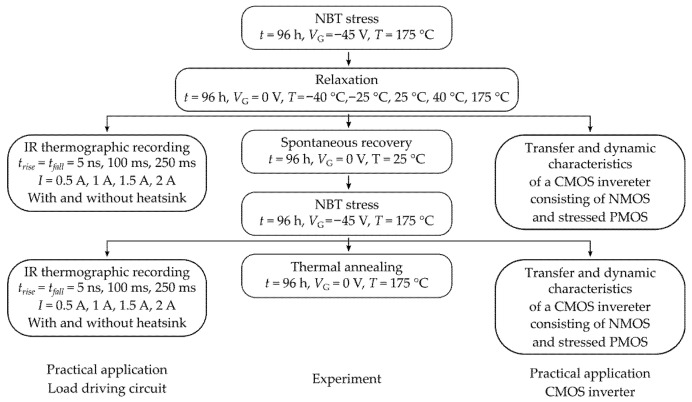
Block schematics of all three lines of the experiment.

A block schematic of the entire experiment is given in Figure 5.

Each of the experimental phases will be explained separately, in more detail, in the following sections.

### 2.1. Stressing

#### 2.1.1. Negative Bias Temperature Stressing

The central line of the experiment is the negative bias temperature stressing of the samples. This part of the experiment has five different phases. The first phase is NBT stressing.

In this phase, samples are subjected to the accelerated NBT stressing conditions. Continuous voltage of −45 V was applied to the gate terminal, while the source and drain terminals were grounded. Stressing was performed in the heating chamber at a temperature of 175 °C for a duration of *t* = 96 h.

**Figure 6:**
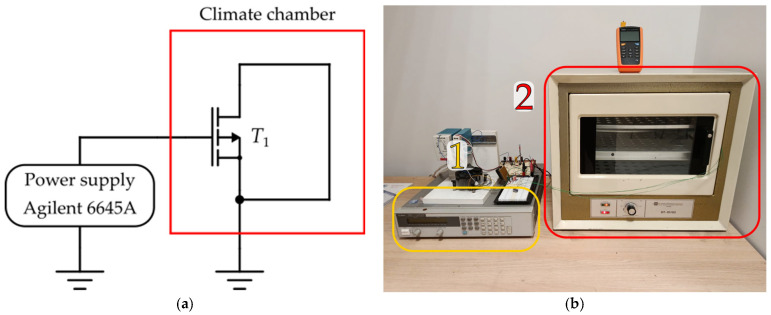
Laboratory setup used for NBT stressing of devices: (**a**) schematic; (**b**) photo during the experiment in the laboratory: (1) Agilent 6645A (Santa Clara, CA, USA) power supply, (2) climate chamber used for temperature control.

Accelerated stressing demands a voltage magnitude of *V*_G_ = −45 V, which is not typical within the generic signal source units. Because of that, a high voltage stress circuit was designed, which is detailly described in our previous paper [33]. The laboratory setup for NBT stressing is shown in Figure 6.

The main measuring equipment is the Keysight B2901A Source Measure Unit (SMU), which is controlled with a laptop, performing measurement of I-V transfer characteristics in the saturation region. All of the measurements are carried out at room temperature, outside the heating chamber. The measured characteristics are obtained with a short-circuited gate and drain terminals. Controlled voltage is sourced from the SMU, and the drain current was measured.

**Figure 7:**
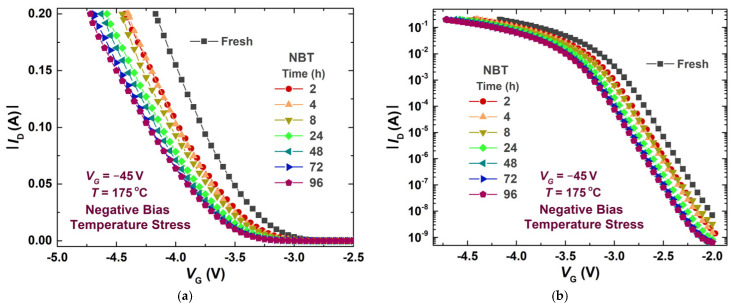
An example of measured transfer characteristics for one of the samples during the first experiment phase (**a**) above threshold transfer characteristics; (**b**) subthreshold transfer characteristics.

The measurement procedure was repeated for all samples at each phase of the experiment according to predefined time intervals. The I-V transfer characteristics in the saturation region, presented in Figure 7, are used to determine the threshold voltage values.

The experimental methodology adopted in this work is based on long-term NBT stressing at room temperature, measuring the transfer characteristics, which is commonly used in reliability studies of power VDMOS transistors operating under high gate voltages and elevated temperatures. Following similar approach as proposed in the JEDEC procedure, which is primarily defined for planar p-channel MOSFETs and focuses on shorter stress times and specific measure–stress–measure or in vivo measurement sequences. Also, this standard has recently been superseded by the updated JESD241 standard, which focuses on advanced CMOS technologies and scaled devices. Consequently, some research groups did similar methodological adaptations for their research [34]. Our approach is modified for commercial TO-220 power devices and is intended to emulate realistic long-duration operating conditions in practical applications. Following a similar approach as proposed in the JEDEC procedure, it provides additional insights into the combined influence of extended stress duration, wide temperature range and subsequent relaxation/annealing phases on the threshold voltage shift and the behavior of complete circuits such as load drivers and CMOS inverters.

#### 2.1.2. Relaxation

The second phase is relaxation. During this phase, no bias is applied to the samples, but the samples stay inside the heating chamber. Here, the samples are divided into several groups. Part of the samples are subjected to the temperature of 175 °C, 40 °C, 25 °C, −25 °C and −40 °C. Additional attention should be given to the behavior of devices operating in below-zero temperatures. While the application range of the VDMOS devices also covers many cases where the devices and their comprising circuits operate in temperatures below 0 °C, there are not many studies investigating reliability under these conditions. Because of that, a part of this experiment was dedicated to analyzing the impact of below-zero temperatures on the behavior and reliability of VDMOS devices.

Constant temperatures of −25 °C and −40 °C were maintained using the climate chamber BINDER MKF 56 (Shanghai Jianheng Instrument Ltd., Shanghai, China), while the other temperatures were maintained using a standard heating chamber. The duration of relaxation is also *t* = 96 h, and transfer characteristics in the saturation region are measured in an identical manner to the first phase of the experiment.

After the second phase of the experiment, some samples are used for IR thermographic recordings, some for CMOS inverter circuit characteristics, and some for the NBTS experiment.

#### 2.1.3. Continuation of the Experiment

The third phase of the experiment involves spontaneous recovery with no bias, and no direct temperature change is performed. Samples are kept at a room temperature of 25 °C.

The fourth phase of the experiment is the second cycle of NBT stressing. Both the stressing procedure and the measuring procedure are identical to those in the first phase of the experiment (*V*_G_, *T*, *t*), as explained in Section 2.1.1. After the fourth phase, some samples are used for IR thermographic recordings and CMOS inverter circuit characteristics measurement, while other samples are subjected to the fifth phase of the experiment.

The fifth phase of the experiment is thermal annealing at the elevated temperature of 175 °C without bias applied. Measuring is performed at room temperature using an identical routine to that in the previous phases of the experiment. A tabular presentation of the used samples classification is given in Table 1.

### 2.2. Infra-Red Thermographic Recordings

**Figure 8:**
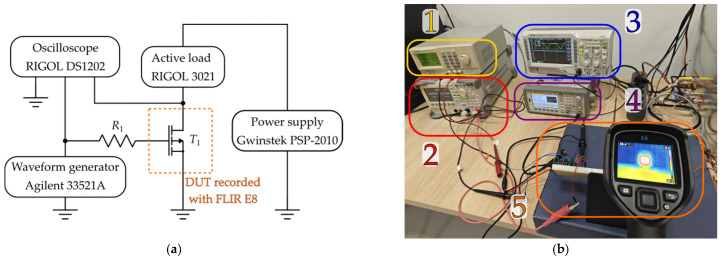
Measurement setup used to measure transistor self-heating: (**a**) schematic; (**b**) photo during measurement in the laboratory: (1) Gwinstek PSP-2010 (Taipei, Taiwan) power supply, (2) RIGOL DL3021A (Beijing, China) active load, (3) RIGOL DS1202 (Beijing, China) oscilloscope, (4) Agilent 33521A (Santa Clara, CA, USA) arbitrary waveform generator, (5) FLIR E8 thermal camera (Wilsonville, OR, USA) recording device used for test.

The second line of the experiment involves the investigation of the self-heating effects evaluated using infra-red thermographic recordings. To examine the thermal behavior of the tested p-channel VDMOS transistors under different operating conditions, infra-red thermographic measurements were performed using a FLIR E8 thermal camera. The experimental arrangement, shown in Figure 8, was designed to emulate realistic operating scenarios in which power VDMOS transistors are commonly used. In such applications, devices are frequently exposed to rapid transitions between on/off-states and thermally demanding environments.

**Figure 9:**
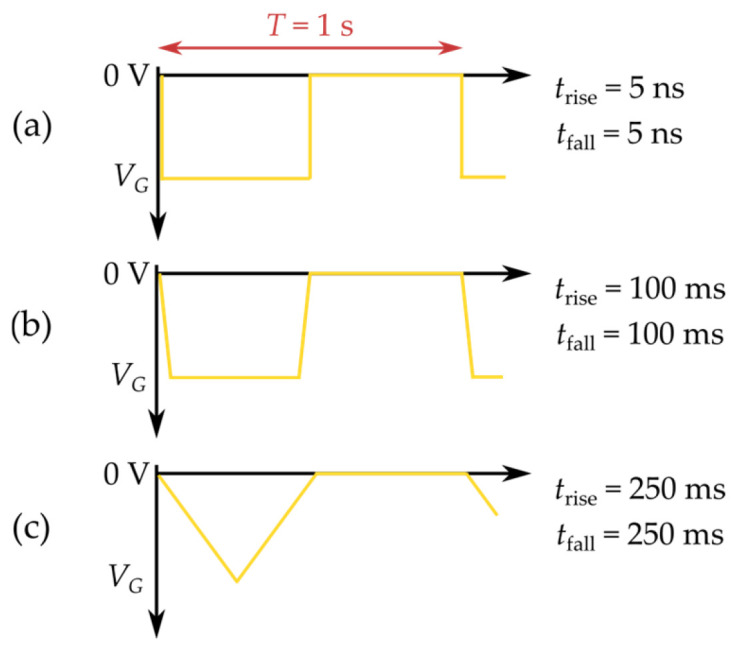
Controlling signals used in the experiment (*f* = 1 Hz, *V*_G_ = 10 V): (**a**) *t*_rise_ = *t*_fall_ = 5 ns; (**b**) *t*_rise_ = *t*_fall_ = 100 ms; (**c**) *t*_rise_ = *t*_fall_ = 250 ms.

In this experiment, the drain terminal was connected to a DC supply voltage, while the transistor gate was driven by pulsed signals that replicate typical switching conditions. Three different control waveforms, illustrated in Figure 9, were applied, each defined by distinct rise and fall times (5 ns, 100 ms and 250 ms) at a frequency of 1 Hz, amplitude of −10 V and a 50% duty cycle [25]. The test signals were generated using an Agilent 33921A (Santa Clara, CA, USA) arbitrary waveform generator. A Rigol DS1202 oscilloscope was used to enable accurate waveform delivery to the device gate.

To evaluate the impact of different load conditions, a Rigol DL3021 active load was connected to the drain circuit and configured to draw four different constant current levels: 0.5 A, 1 A, 1.5 A and 2 A. This enabled investigation of the thermal response as a function of power dissipation.

**Figure 10:**
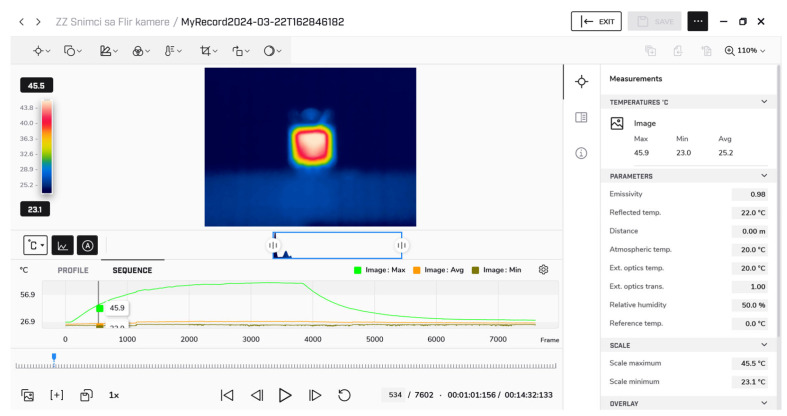
Screenshot of software FLIR Thermal Studio, which was used for extraction of temperature, and setup used to measure transistor self-heating.

The camera was connected to a computer and controlled via dedicated software, FLIR Thermal Studio (version 2024), enabling continuous acquisition of temperature profiles throughout the predefined measurement interval. The interface of the software during the measurement is presented in Figure 10. The image at the center illustrates the levels of the device temperature. Different colors represent different temperature levels, providing a visual indication of how the transistor heats up during operation. This setup provided precise monitoring of the device surface temperature during both heating and cooling phases.

During each test, the transistor was switched on for approximately 6 min (heating phase) and turned off for the following 6 min (cooling phase). This 12 min cycle was selected based on preliminary observations showing that the device temperature reaches a maximum within this interval [5,25]. Temperature evolution was recorded for both fresh samples and devices previously subjected to NBT stress and the subsequent relaxation process (after the second phase of the experiment and after the fourth phase of the experiment), allowing comparison of thermal behavior before and after degradation.

This measurement methodology provides detailed insight into the influence of dynamic switching conditions, gate-signal characteristics, and load variations on the self-heating behavior of VDMOS transistors, which is an essential aspect when analyzing their reliability and long-term stability.

This methodology has previously proven effective for evaluating self-heating effects in power MOSFETs [5,25], and it provides valuable insight into the thermal response of p-channel VDMOS transistors, particularly considering their typically higher on-state resistance and different carrier mobility degradation mechanisms.

### 2.3. Characterization of CMOS Inverter Containing NBT Stressed Device

**Figure 11:**
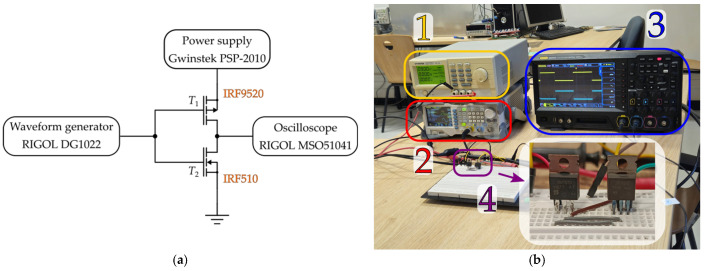
Measurement setup used for CMOS inverter characterization: (**a**) schematic; (**b**) photo during measurement in the laboratory: (1) Gwinstek PSP-2010 (Taipei, Taiwan) power supply, (2) RIGOL DG1022 (Beijing, China) waveform generator, (3) RIGOL DS1202 (Beijing, China) oscilloscope, (4) CMOS inverter circuit designed using n-channel power VDMOSFET IRF510 (Vishay, Malvern, PA, USA) and p-channel power VDMOSFET IRF9520 (Vishay, Malvern, PA, USA) .

The third line of the experiment is focused on eliminating the impact of NBTI on circuit performance, more precisely on the performance of the CMOS inverter containing an NBT-stressed p-channel VDMOSFET. A CMOS inverter is selected as one of the most fundamental logic gates and a part of numerous circuits. It consists of PMOS and NMOS transistors that are connected in a complementary configuration, as shown in Figure 11a.

If the input is at a high voltage level, the NMOS transistors turn on, pulling the output to the low voltage level. On the other hand, if the input is on a low voltage level, the PMOS turns on and pulls the output to the high voltage level. The behavior of the CMOS inverter is typically characterized through transfer and dynamic characteristics [28,29,32]. The transfer characteristic of a CMOS inverter presents the output voltage changes in response to the input voltage, while the dynamic characteristics of a CMOS inverter describe its response to changes over time, including rise time, fall time, and propagation delay.

**Figure 12:**
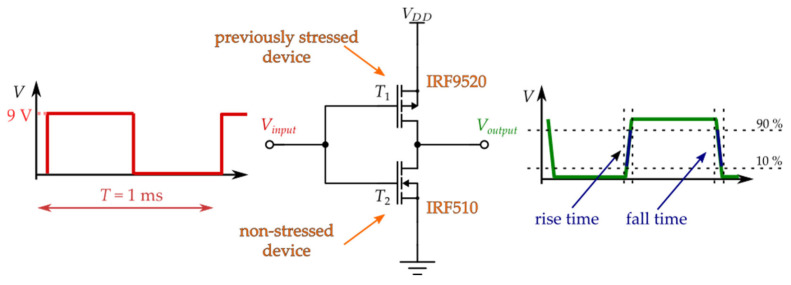
Signal and circuit setup for CMOS inverter characterization.

In this experiment, the behavior of the CMOS inverter that comprises a previously stressed p-channel power VDMOS transistor IRF9520 and a non-stressed n-channel power VDMOS transistor IRF510 was analyzed (illustrated in Figure 12). Commercial n-channel power VDMOSFET IRF510 is selected for the CMOS inverter because of its almost compatible characteristics with IRF9520 [35]. The maximum drain current is 5.6 A, and the threshold voltage is in the identical range, between 2 and 4 V. It is also encapsulated in TO-220 packages with an identical pin order.

**Figure 13:**
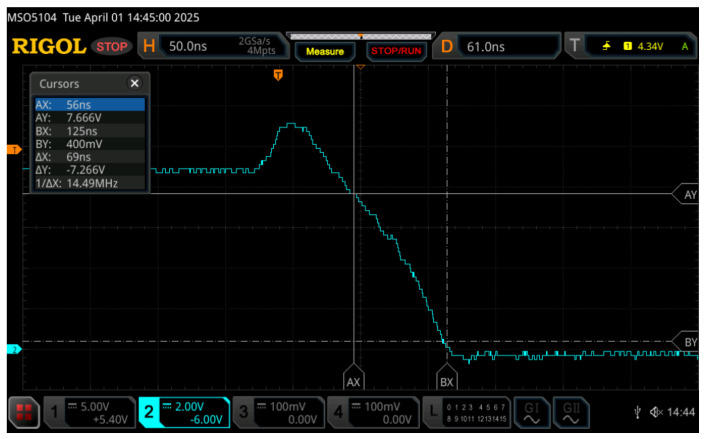
The transition between the high voltage level and the low voltage level captured using an oscilloscope and used to determine the transfer and dynamic characteristics of a CMOS inverter.

Besides these devices, as illustrated in Figure 11, a programmable DC power supply, the GW Instek PSP-2010 (Taipei, Taiwan), was used to provide bias voltages for the transistors. The inverter input square-wave signal was generated by a RIGOL DG1022 waveform generator, while a RIGOL MSO5104 (Beijing, China) oscilloscope was used to capture input and output signal waveforms. The supply voltage for the CMOS inverter was *V*_DD_ = 9 V, while characteristics of the input signal were *V*_HIGH_ = 9 V, *V*_LOW_ = 0 V, *f* = 1 kHz, DTC = 50% and *t*_rise_ = *t*_fall_ = 5 ns. Measuring transfer and dynamic characteristics involves acquiring signal waveforms from the oscilloscope in the form of a .csv file. The sample of the data acquired with the oscilloscope is given in Figure 13.

The data from the captured file is then processed using a specially designed Python script (Python version 3.13) with the goal of obtaining transfer characteristics as well as the *t*_rise_ and *t*_fall_ of the CMOS inverter output signal.

## 3. Results and Discussion

### 3.1. Stress-Induced Threshold Voltage Shift

**Figure 14:**
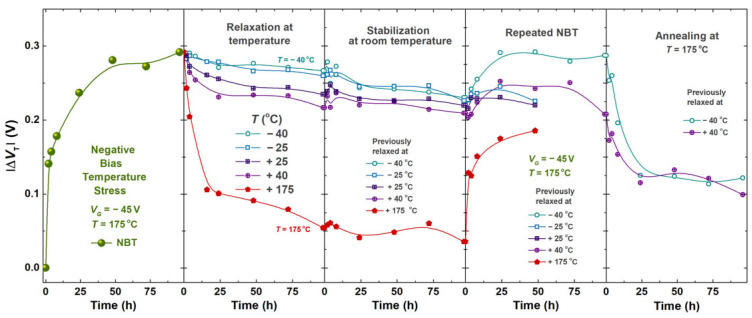
Threshold voltage shifts (average value per group) for all of the samples subjected to different experiment phases presented in Section 2.1.

For every transfer characteristic measured during the experiment, the threshold voltage value is determined. The threshold voltage shifts throughout the experiment phases for all of the groups of devices are presented in Figure 14.

Negative bias temperature (NBT) stress has a well-established impact on charge trapping mechanisms within the gate oxide and at the oxide–semiconductor interface. These processes directly influence the electrical behavior of MOSFETs, where the most notable change is the increase in the value of the threshold voltage, as can be seen in the first part of Figure 14. In p-channel devices, prolonged NBT stress promotes the trapping of positive charge in the oxide and the capture of channel holes at interface traps. Both mechanisms effectively act against the applied gate voltage, resulting in an increased magnitude of the threshold voltage [5,21,36].

During the NBT stress, *V*_T_ varies, which naturally suggests the involvement of different underlying degradation mechanisms. The increase in positively charged oxide charge under high negative oxide fields and elevated temperatures can be attributed to hole trapping and oxygen vacancy-related defects. These processes may proceed through reactions such as

(1) $$O_{3}\equiv {\mathrm{Si}}^{••}\mathrm{Si}\equiv O_{3}+h^{+}\to O_{3}\equiv {\mathrm{Si}}^{+•}\mathrm{Si}\equiv O_{3}$$

Also, this could happen at the weakened Si–H bonds near the SiOi_2_/Si interface.

Similarly, the buildup of interface traps *N*_it_ observed during NBT stressing resulted from the combined action of the strong electric field and elevated temperature, which enables the dissociation of Si–H bonds at the SiO_2_/Si interface:

(2) $${\mathrm{Si}}_{3}\equiv \mathrm{Si}-H↔{\mathrm{Si}}_{3}\equiv {\mathrm{Si}}^{•}+H^{•}$$

The released hydrogen species are highly reactive and may further contribute to interface degradation by breaking additional interface Si–H bonds, enabling the formation of new interface traps.

Following the NBT stress phase, the devices undergo a relaxation period during which partial recovery of electrical parameters is expected, as can be seen in the second part of Figure 14. Higher temperatures generally accelerate detrapping processes and thus enhance recovery. However, the experimental findings show that the threshold voltage does not fully return to its initial value, indicating the presence of permanent degradation.

The third part of Figure 14 presents threshold voltage shifts during relaxation at room temperature without biasing, when the spontaneous recovery occurs. Relaxation at room temperature exhibits only a minor influence on the previously stressed device, confirming that thermal activation is essential for effective recovery. Still, the change during this phase is the most pronounced with the group of samples previously relaxed at the lowest temperatures (−40 °C and −25 °C). Room temperature (25 °C) is a significant increase in the temperature for these groups of samples, leading to thermal activation-induced recovery.

The second NBT stress cycle produces only a modest additional threshold voltage shift, as can be seen in the fourth part of Figure 14. These results reinforce the conclusion that the degradation mechanisms approach saturation after the initial stress, since the total increment of the NBT stress-induced threshold voltage shift during the second cycle is less pronounced.

The last experiment phase, thermal annealing at 175 °C, leads again to the partial recovery of the NBT stress-induced threshold voltage shift, presented in the fifth part of Figure 14. Still, similar to the relaxation phase, recovery seems to saturate after a specific time. Since all samples are annealed at the same annealing temperature, the magnitude and shape of the curve of the threshold voltage shift during this phase are similar for all groups of devices, regardless of previous treatment. This behavior is consistent with the strong temperature dependence of detrapping processes.

### 3.2. Self-Heating in Practical Applications

**Figure 15:**
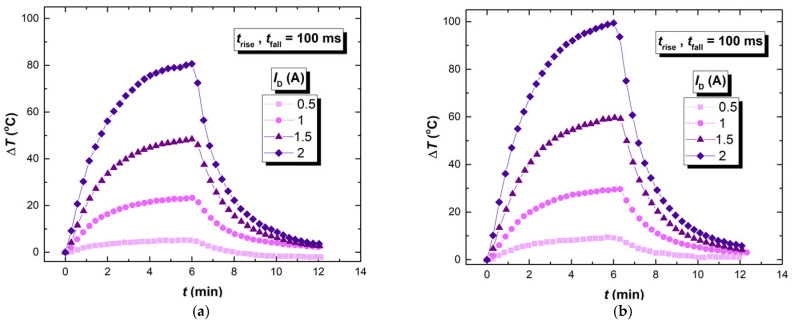
Thermal response of devices to a trapezoidal gate-controlling signal (*t*_rise_ = *t*_fall_ = 100 ms) at four different drain current levels 0.5 A, 1 A, 1.5 A and 2 A: (**a**) D12 (NBT stressed and relaxed at −40 °C); (**b**) D15 (NBT stressed, relaxed at −40 °C, relaxed at room temperature and again NBT stressed).

The results of the infra-red thermographic recording can be observed through three comparisons. The first one is the comparison of the devices’ self-heating when the same controlling signal waveform is applied for different drain current values. The second one is the comparison when the same drain current value is applied using the different controlling signal waveforms, while the third one is focused on how the previous treatment of the samples (different experiment groups) affects self-heating. Figure 15 shows the temperature response of the devices to a trapezoidal gate-controlling signal (*t*_rise_ = *t*_fall_ = 100 ms) at four different drain current levels (0.5 A, 1 A, 1.5 A and 2 A). The temperature response of the device after the first two experiment phases (D12 from Table 1) is presented in Figure 15a, while the temperature response of the devices after the first four experiment phases (D15 from Table 1) is presented in Figure 15b.

In Figure 15, the self-heating temperature, given as a difference between the measured temperature and the room temperature, is presented as a function of time (*t*). A consistent trend was observed across all measurement conditions. The higher value of the drain current resulted in more pronounced self-heating. The highest device temperature is accomplished with the 2 A drain current, while the lowest device temperature is accomplished with the 0.5 A drain current.

The most pronounced increase in the device temperature, for all of the analyzed groups of samples, is shown within the first two and a half minutes of the experiment, where the temperature increase rate reached its maximum.

Compared to the previously non-stressed device, NBT stressed devices exhibited greater self-heating. In Figure 15b, the self-heating is even more pronounced, since the device group D15 went through the identical first two phases of the experiment like group D12 (NBT stress and relaxation at 175 °C), but also two additional phases, room temperature relaxation and an additional cycle of NBT stressing. Very similar behavior is also noticed within the other pairs of device groups subjected to the thermographic recording when the first two experiment phases are identical, after which one group proceeds to additional treatment (D6 and D7, D9 and D10). These results point out the direct impact of NBT stressing and relaxation on this type of device application. After the devices were switched off, the temperature of the devices gradually decreased and spontaneous cooling occurred. For all devices and drain current values, a similar shape of the cooling curve is observed. More details can be given with the comparison of the heating and cooling rate values, as given in Table 2.

Values of the initial heating and cooling rates are significantly higher than the average ones. The relation between the initial and the average heating rate is similar for all of the drain currents, with the initial being approximately three times greater than the average heating rate. The results for the device group D12 presented in Table 2 are quantitatively and qualitatively in line with the results obtained for other device groups subjected to thermographic recording.

Self-heating of the devices when driven with different controlling signal waveforms needs to be analyzed in more detail. Total self-heating recorded in devices is a combination of two effects. The first effect is the heating caused by power dissipation when the transistor is turned on and in its current-conducting state. As the interval during which the transistor is turned on becomes longer, this type of self-heating becomes more pronounced. The second effect is the increase in the self-heating caused by the power dissipation, where the transistor switches states between on and off.

While the first effect is not directly related to the consequences of the NBT stressing, the second effect is directly related to the change in the *R*_ch_, which is increased because of the NBT stressing. Still, the magnitude of impact of this effect is dependent on the switching frequency, duty cycle and the rise and fall times of the controlling signal [37]. For lower frequencies, with shorter transient times, conductive self-heating will be dominant over the transient self-heating, meaning that, on a larger scale, transient self-heating could be negligible. But, during high-frequency switching, which is present in many VDMOS transistor applications, transient self-heating effects become relevant. Similar studies show that this type of SHE can be more emphasized than the active SHE for specific working conditions [25]. Therefore, in this case, it is not possible to give clear conclusions, unlike in the case of Figure 15, since the change in the self-heating is caused by multiple factors.

In many real-world practical applications of p-channel power VDMOSFET, these devices are used connected to a heatsink. Application of the heatsink is typical for power devices, including power VDMOSFETs, since power devices are designed to conduct higher values of currents and tend to suffer from heating. In order to examine this type of practical application, additional rounds of IR thermographic recordings have been carried out under groups of devices D15 with two different heatsinks.

**Figure 16:**
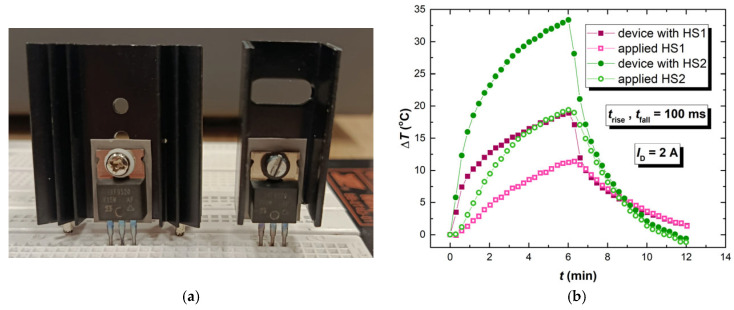
(**a**) IRF9520 transistor with mounted HS1 [38] and with mounted HS2 [39]; (**b**) Temperature response of the device group D15 with mounted HS1, of HS1, with mounted HS2 and HS2, operating with the drain current of 2 A and with *t*_rise_ = *t*_fall_ = 100 ms gate-controlling signal.

The first heatsink (HS1) is an extruded aluminum heatsink, SK 104 50.8 STS, manufactured by Fischer Elektronik (Lüdenscheid, Germany) [38], while the other smaller heatsink (HS2) is also an aluminum heatsink, Alutronic PR17/35II/SE (Halver, Germany) [39]. Both of the heatsinks are compatible with the TO-220 package. Figure 16a presents samples with mounted HS1 and HS2. IR thermographic recording was performed four times using the routine presented in Section 2.2. For both of the heatsinks, recording was performed twice: first, recording the device temperature, and second, recording the heatsink temperature. Figure 16b presents the temperature response of the device group D15 with mounted HS1 and with mounted HS2, operating with a drain current of 2 A and a *t*_rise_ = *t*_fall_ = 100 ms gate-controlling signal.

As can be seen from Figure 16b, the application of a heatsink drastically reduced the temperature of the device. Even with the smaller heatsink (HS2), device temperature does not rise beyond 35 °C, whereas without the heatsink, device temperature exceeds 80 °C. With the usage of an appropriate heatsink, the negative effects of prior NBT treatment can be overcome or reduced.

### 3.3. Impact of Stressing on the CMOS Inverter Circuit Performance

**Figure 17:**
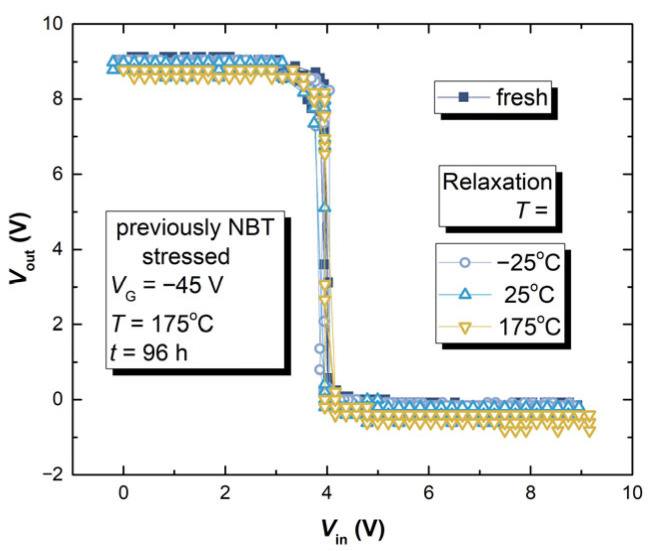
Transfer characteristics of a CMOS inverter designed using a previously stressed p-channel power VDMOSFET (D1, D6, D9) and a non-stressed n-channel power VDMOSFET.

Figure 17 presents transfer characteristics of a CMOS inverter designed using a previously stressed p-channel power VDMOSFET (D1, D6, D9) and a non-stressed n-channel power VDMOSFET. It is worth mentioning that each oscilloscope captured a very large number of points (10^6^). During results processing, the number of presented data points is reduced in order to improve clarity and visibility.

**Figure 18:**
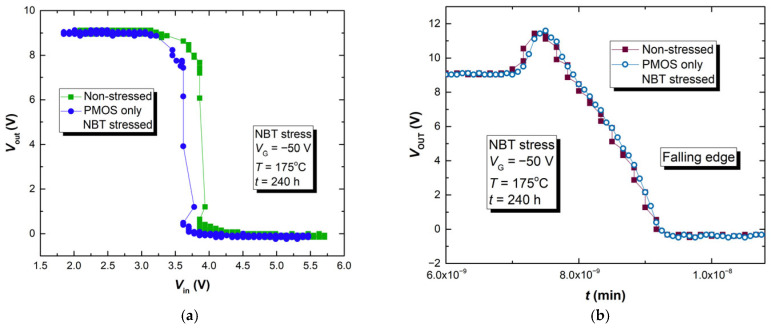
The results of the CMOS inverter built with a ten-day NBT-stressed p-channel VDMOSFET IRF9520 and a non-stressed n-channel VDMOSFET IRF510: (**a**) transfer characteristics; (**b**) falling edge.

As can be seen from Figure 17, the impact of NBT stressing followed by relaxation does not affect CMOS inverter transfer characteristics drastically. Similar behavior is noted for the groups of devices, D2, D7 and, D10 that passed two additional experiment phases. From these results, it was not possible to clearly examine the impact of NBT stressing on the performance of the CMOS inverter circuit. Therefore, an additional experiment has been carried out where p-channel VDMOS devices were subjected to the more intense NBT stress (*V*_G_ = −50 V, *T* = 175 °C, the critical conditions from Figure 1) in a manner explained in Section 2.1.1, but for a longer period of time (240 h instead of 96 h). The measured transfer characteristics of the CMOS inverter built with p-channel VDMOSFETs that passed this treatment and non-stressed n-channel VDMOSFET are presented in Figure 18.

The transfer characteristics of a CMOS inverter with a p-channel device that was NBT-stressed for ten days show a clear shift to the left. During the ten days of NBT stressing, the threshold voltage value of the p-channel VDMOSFET shifted enough so that it could affect the transfer characteristic of a CMOS inverter. Similar results can be seen from Figure 18b, where the dynamic characteristics, more precisely the fall time, are compared. The fall time increased, and similar results are obtained for the rise time. It can be concluded that, under harsher operating conditions, NBT stress-induced threshold voltage shift in the p-channel power VDMOSFET presents additional deterioration of the circuit operation.

However, for the analysis of the circuit performance, it is important to analyze one additional factor. From the standpoint of practical application, it would be rare to use a circuit where only a single transistor is stressed, while the other components in the circuit are completely fresh. Ambient conditions usually affect the entire circuit, whereas some of the devices are more susceptible to the environmental conditions than others. Therefore, an additional round of experiments regarding the examination of the NBTI on the circuit performance has been carried out. Special attention is given to the analysis of the circuit’s performance over a broad temperature range.

**Figure 19:**
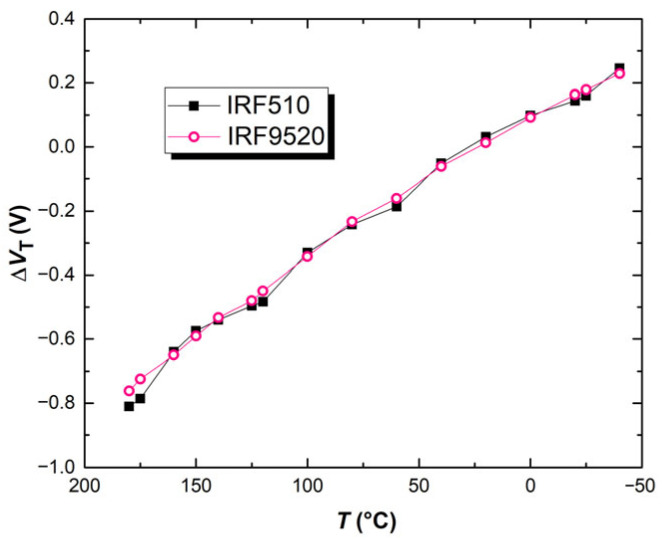
Temperature dependence of *V*_T_ for p-channel VDMOSFET IRF9520 and n-channel VDMOSFET IRF510, which comprise the analyzed CMOS inverter.

First, thermal characterization of both the p-channel power VDMOSFET IRF9520 and n-channel power VDMOSFET IRF510 was performed using the BINDER MKF 56 climate chamber. Transfer characteristics in the saturation region at different temperatures in the range from −40 to 180 °C, at 20 °C intervals, were measured. The threshold voltage value was determined for each of the measured characteristics. The temperature shift in the threshold voltage for both of the analyzed devices is presented in Figure 19.

The average value of the threshold voltage shift on the observed temperature range, calculated from the results presented in Figure 19, for the p-channel VDMOSFET IRF9520 is 4.5 mV/°C, while for the n-channel VDMOSFET IRF510, it is 4.8 mV/°C. These devices show a similar temperature-induced threshold voltage shift, suggesting that the circuit containing the analyzed devices should exhibit stability at elevated temperatures.

**Figure 20:**
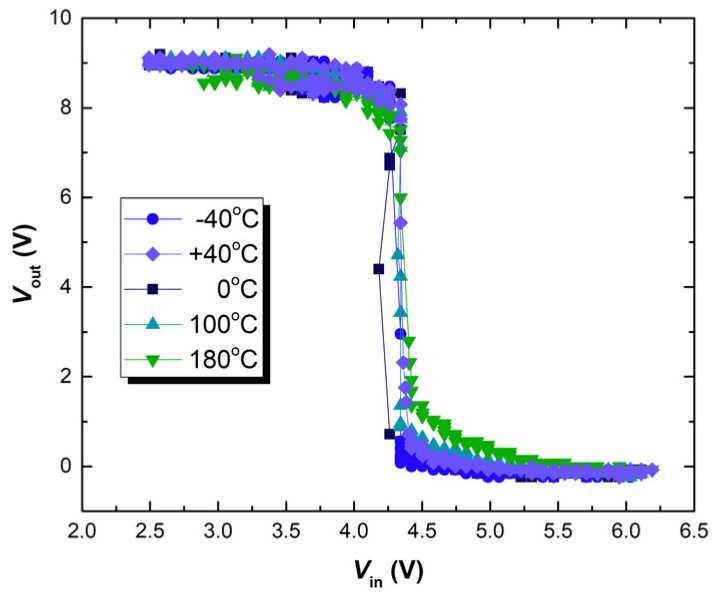
Determined transfer characteristics of the CMOS inverter circuit under different temperatures during the additional experiment.

Therefore, a circuit with a CMOS inverter consisting of analyzed devices was put into the BINDER MKF 56 climate chamber in order to perform thermal characterization on a circuit level. While in the climate chamber, the circuit of the CMOS inverter remains connected to the measuring setup in an identical manner, as shown in Figure 11. Thermal characterization was performed in an identical temperature range for the single samples (from −40 °C to 180 °C), while the dynamic and transient characteristics were measured at 20 °C intervals. The determined transfer characteristics of the CMOS inverter circuit under temperatures of −40 °C, 0 °C, 100 °C and 180 °C during the additional experiment are presented in Figure 20.

**Figure 21:**
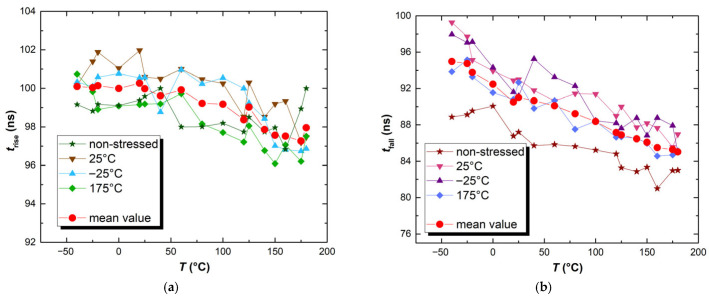
Comparison of rise (**a**) and fall (**b**) times for output CMOS inverter circuits designed with previously stressed p-channel devices and non-stressed n-channel devices, at different temperatures.

In the temperature range above 40 °C, a clear shift in the transfer characteristics to the right is noted for all of the analyzed samples. As can be seen for the characteristics measured at −40 °C and at 0 °C, there is no distinct change between the determined results in the lower temperature ranges. In the range from −40 °C to +40 °C, as can be seen from Figure 19, the temperature-induced threshold voltage shift is not drastic. In accordance with the results from Figure 17, the threshold voltage shift needs to be more pronounced in order to affect the transfer characteristics of the CMOS inverter circuit. When the threshold voltage shifts in the transistors comprising the CMOS inverter circuit become more pronounced (at temperatures above +40 °C), a constant significant shift in the transfer characteristics can be noted. Similar considerations can be given for the dynamic characteristics of the CMOS inverter circuit, as can be seen from Figure 21.

Generally, in the CMOS inverter circuit, the fall time is shorter than the rise time. This is caused by the greater mobility of the electrons, which are charge carriers in the n-channel device, when compared with holes, which are charge carriers in the p-channel devices. The results presented in Figure 21 show agreement with the previous reports in that manner [28,31,32]. The threshold-voltage shift in the p-channel device results in a significant mismatch between the p- and n-channel threshold voltages within the CMOS inverter. During the fall time (transition from high voltage level to low voltage level), the p-channel is turning off, and the n-channel device is turning on. Since the absolute value of the threshold voltage of the p-channel device is increased, the p-channel device will start turning off before the n-channel device starts turning on. Therefore, more time is needed to perform the transition, demonstrating that the impact of stressing is far more pronounced during the fall time, presented in Figure 21b, than the rise time, presented in Figure 21a. This is a direct impact of the NBT stress-induced threshold voltage shift on the performance of the CMOS inverter circuit. It is interesting that although the four groups of devices shown in Figure 21 have undergone distinct prior treatments, some tendencies could be noted. When examining the mean value of the rise time, it can be seen that, up to approximately 40 °C, all components maintain almost a constant value. However, above 40 °C, a noticeable decrease in rise time is observed as the temperature increases. Analyzing Figure 21b, it can be observed that over the entire investigated temperature range, the fall time exhibits a decreasing trend.

Because of the described effects, the difference between the rise and fall times at elevated temperatures is more pronounced. For example, for a CMOS inverter circuit containing D1 that was NBT-stressed and relaxed at 175 °C, the rise time and fall time at 0 °C are 99.3 ns and 91.6 ns, respectively (a difference of 7.7 ns). An identical circuit, at 100 °C, shows a rise time of 97.9 ns, while the fall time is 88.2 ns (a difference of 9.7 ns), which is an increase of more than 20%. It can be concluded that the impact of stressing on the performance of the CMOS inverter circuits increases with the increase in the operating temperature.

## 4. Conclusions

In this paper, the impact of NBTI on commercial power p-channel VDMOS transistors IRF9520 in practical applications of a load driver circuit and a CMOS inverter circuit is examined. NBT stress induces a threshold voltage shift that is more pronounced with higher gate voltages and higher temperatures. Pulsed NBT stress-induced threshold voltage shift is shown to be commonly lower than in the case of static NBT stressing because of the partial recovery of degradation that occurs during pulsed NBT stressing.

The induced threshold voltage shift during pulsed stressing is also shown to be dependent on the characteristics of the pulsed gate signal, such as amplitude, frequency and the duty cycle, pointing out that these gate signal characteristics affect practical applications of the devices. Various forms of stress are applied to the samples with the goal of emulating real-world conditions of operation. The stressing and measuring process in each part of the experiment is explained in detail.

The results suggest that NBT stressing directly affects the examined samples operating in the load driving circuit through increased self-heating. For all of the tested samples, self-heating during operation is increased in value, corresponding to the NBT stressing and relaxation-induced threshold voltage shift. Increased self-heating could be partially mitigated with the use of a heatsink. Future research should be directed to examining how self-heating affects the signal rise and fall times of the gate-controlling signal.

The results also suggest that NBT stressing directly affects the characteristics of the CMOS inverter comprising the examined samples. An impact is seen with the shift in transfer characteristics and increase in transient times, and it is far more pronounced at elevated temperatures during operation. Future work should focus on investigating the dependence of the input signal frequency on the characteristics of the CMOS inverter containing an NBT-stressed p-channel device and the impact on more complex circuits.

## Figures and Tables

**Table 1:** Groups of devices under test and treatment of each group of devices.

	D1	D2	D3	D4	D5	D6	D7	D8	D9	D10	D11	D12	D13	D14	D15
NBTS, *t* = 96 h *V*_G_ = −45 V, *T* = 175 °C	X	X	X	X	X	X	X	X	X	X	X	X	X	X	X
Relaxation, *t* = 96 h *T* = 175 °C	X	X	X												
Relaxation, *t* = 96 h *T* = 40 °C				X	X										
Relaxation, *t* = 96 h *T* = 25 °C						X	X	X							
Relaxation, *t* = 96 h *T* = −25 °C									X	X	X				
Relaxation, *t* = 96 h *T* = −40 °C												X	X	X	X
IR and CMOS	X			X		X			X			X			
Spont. Recovery, *t* = 96 h, *T* = 25 °C		X	X		X		X	X		X	X		X	X	X
NBTS, *t* = 96 h *V*_G_ = −45 V, *T* = 175 °C		X	X		X		X	X		X	X		X	X	X
IR and CMOS		X					X			X					X
Annealing, *t* = 96 h *T* = 175 °C			X		X			X			X		X		

**Table 2:** Heating rate for samples NBT stressed (*V*_G_ = −45 V, *T* = 175 °C) and relaxed at −40 °C (D12).

Drain Current [A]	Initial Rates (First 60 s) [°C/min]		Average Rates [°C/min]	
	Heating	Cooling	Heating	Cooling
0.5	2.42	3.39	0.87	0.52
1 A	11.2	10.92	3.88	3.50
1.5 A	23.00	22.77	8.06	7.62
2 A	39.12	43.04	13.43	12.83

## Data Availability

The original contributions presented in the study are included in the article; further inquiries can be directed to the corresponding author.
